# Genomics, Big Data, and Broad Consent: a New Ethics Frontier for Prevention Science

**DOI:** 10.1007/s11121-018-0944-z

**Published:** 2018-08-25

**Authors:** Celia B. Fisher, Deborah M. Layman

**Affiliations:** Department of Psychology, Fordham University, Dealy Hall 441, East Fordham Road, Bronx, NY 10458 USA

**Keywords:** Informed consent, Broad consent, Genetics, Prevention science, Privacy, Research ethics

## Abstract

Emerging technologies for analyzing biospecimens have led to advances in understanding the interacting role of genetics and environment on development and individual responsivity to prevention and intervention programs. The scientific study of gene-environment influences has also benefited from the growth of Big Data tools that allow linking genomic data to health, educational, and other information stored in large integrated datasets. These advances have created a new frontier of ethical challenges for scientists as they collect, store, or engage in secondary use of potentially identifiable information and biospecimens. To address challenges arising from technological advances and the expanding contexts in which potentially identifiable information and biospecimens are collected and stored, the Office of Human Research Protections has revised federal regulations for the protection of human subjects. The revised regulations create a new format, content, and transparency requirements for informed consent, including a new mechanism known as broad consent. Broad consent offers participants a range of choices regarding consent for the storage and future use of their personally identifiable data. These regulations have important implications for how prevention scientists and oversight boards acquire participant consent for the collection, storage, and future use of their data by other investigators for scientific purposes significantly different from the original study. This article describes regulatory changes and challenges affecting traditional informed consent for prevention research, followed by a description of the rationale and requirements for obtaining broad consent, and concludes with a discussion of future challenges involving ongoing transparency and protections for participants and their communities.

Emerging technologies for the collection and analysis of biospecimens have led to advances in understanding the interacting role of genetics and environment on the development of mental health and behavioral risk and resilience and individual responsivity to prevention and intervention programs (Dick et al. 2011; Fisher and McCarthy 2013; Musci and Schlomer 2018). The proliferation of genomic research is fueled by the ease of collecting DNA through cheek swabs or saliva, the decreasing costs of genotyping and DNA sequencing (Dick et al. 2011; Lunshof et al. 2008) and technological advances in genome-wide association studies (GWAS) (Calvin et al. 2012; Davies et al. 2016; Greven et al. 2009; Krapohl et al. 2014; Mandelli and Serretti 2013;McCrory et al. 2010; Rimfeld et al. 2015), gene by environment studies (GxE) (Belsky and Pluess 2009; Kegel et al. 2011), and gene by intervention studies (GxI) (Dick et al. 2011; Musci and Schlomer 2018). As a result, the growing field of genomics research has enriched the contributions of prevention science for understanding the unique and interacting roles of genetic and environmental factors in areas including academic achievement, sexual risk behaviors, substance use, internalizing and externalizing disorders, and responsivity to educational and development promoting preventive interventions within the growing field of implementation science (Bakermans-Kranenburg et al. 2008; Beach et al. 2018; Brody et al. 2009; Dick et al. 2011; Fisher 2017b; Glenn et al. 2018; Haworth and Plomin 2012; Leadbeater et al. 2018; Musci and Schlomer 2018; Russell et al. 2017; Zheng et al. 2018).

The progress in utilizing biospecimens in prevention science parallels the rise in integrating large datasets across all scientific disciplines. This trend, sometimes referred to as “Big Data,” is marked by increased access to and use of individual-level data across administrative data systems with the potential to link genomic data to health, public benefits, child welfare, criminal and juvenile justice, and educational records and other personal information (Caspi et al. 2017; Caspi et al. 2002; Perlman and Fantuzzo 2013; Wertz et al. 2018). The accumulation of personal information into large centralized datasets increases opportunities for secondary widespread and ongoing use by diverse investigators (Gilmore 2016; Kaplan et al. 2014).

Along with the scientific benefits, advances in genomic and Big Data analytic tools is the increasing ability of investigators to re-identify previously de-identified participant information (Hansson et al. 2016; Malin and Sweeney 2001). To adapt to ongoing technological advances, research aims, and expanding contexts in which biospecimens are collected, stored, and available for secondary use, the Office of Human Research Protections (OHRP 2017) has issued revised federal regulations for the protection of human subjects (known as the Final Rule) that include expanded requirements for obtaining informed consent in general and a new category known as broad consent. Broad consent requires investigators to offer participants a range of choices regarding consent to the ongoing storage and future use of their personally identifiable data. The revised guidelines, scheduled to go into effect in 2019, have important implications for how prevention scientists will obtain initial consent for research involving collection of biospecimens as well as consent for future use.

This article begins with a discussion of changes to federal regulations on the format, content, and transparency of informed consent across all aspects of prevention science, followed by a description of the rationale, requirements, and implications for obtaining broad consent for the storage and future use of potentially identifiable information and biospecimens. We conclude with a discussion of future challenges that will be raised involving ongoing transparency and protections for participants and their communities.

## Changes in Federal Regulations Governing Key Elements of Informed Consent

The Final Rule is intended to modernize federal regulations for human subjects’ protections to be in step with the genomic revolution, the rapidly changing Big Data technology, and perceived inefficiencies and gaps in protecting the rights of prospective participants (Emanuel and Menikoff 2011; Sugarman 2017). In federal regulations, participant autonomy in deciding whether to participate in a research study is protected through the informed consent process with three key preconditions: information disclosure, participant comprehension, and voluntariness (Sreenivasan 2003; Strauss 2017). However, according to many of the comments elicited during consideration of the federal rule changes, the increasing length and complexity of modern-day consent forms threaten participant autonomy by sacrificing the clarity needed for comprehension in favor of protecting the liability of institutions (Klitzman 2013). A meta-analysis of informed consent in clinical trials found comprehension difficulties for important components including randomization, placebo, and participation risks and benefits (Tam et al. 2015). The revisions to federal regulations were designed to address these problems through new requirements for the format and content of informed consent.

### Format Changes: a Concise Summary of Key Information

In response to lengthy consent forms that often hinder an informed participation decision, the revised regulations emphasize the need to improve the quality and transparency of informed consent. This effort is consistent with recommendations of the Society for Prevention Research (SPR) Ethics Task Force (Leadbeater et al. 2018) underscoring the need for prevention scientist to respect the rights of those whose lives they hope to improve and empower them to make decisions concerning issues that affect them. This refocus on comprehension requires informed consent documents that “as a whole must present information in sufficient detail relating to the research, and must be organized and presented in a way that does not merely provide lists of isolated facts, but rather facilitates the prospective subject’s or legally authorized representative’s understanding of the reasons why one might or might not want to participate” (§__.116 (2)(ii) (OHRP 2017, pp. 7265–7266). To fulfill this aim, the Final Rule requires prior to the full consent form an initial concise summary of key information necessary for a prospective participant to make a decision. Topics covered in this initial summary are similar to requirements for the full informed consent, e.g., purpose, duration of participation, procedures, risks/discomforts, benefits, and alternative procedures/treatment. It is not intended to replace any of the required information or detailed information about procedures and risks but, rather, to highlight the most critical information that may be buried in a lengthy, cumbersome document (Menikoff et al. 2017; OHRP 2017).

Guidance for developing this concise summary and a more comprehensible informed consent is left to what a “reasonable person would want to have in order to make an informed decision about whether to participate” (§__.116 (4) (OHRP 2017, p. 7265). The use of “reasonable person” is intended to increase comprehension and transparency across participant populations and as described later in this article is also applied to requirements for broad consent.

However, requirements for the length and specific content are explicitly left to the discretion of Institutional Review Boards (IRBs) with only minor direction regarding the underlying purpose and rationale. In attempting to address this requirement, investigators and IRBs should consider that different participant populations will have distinct levels of genetic literacy and informational needs and be wary of a scripted approach to the concise consent summary that may ultimately fail to adequately guide prospective participants through decision-making (Condit 2010; Fisher 2017a; Fisher and Wallace 2000).

#### Suggested Key Elements for Consent

A reasonable consent decision for research involving genetic testing may require understanding that the rapid rate at which new genetic technologies develop and the fact that many genes are related to more than one trait (pleiotropy) means that investigators may discover genetic risk that is unanticipated or incidental to the original aims of the research (Cooper et al. 2006). Relatedly, the multifactorial and probabilistic nature of data acquired through collection of genetic information for prevention studies and the lack of clinical utility can confuse participants attempting to understand the personal relevance of research results, leading to unrealistic expectations regarding the possibility of direct benefits (Fisher 2017b; Henderson 2008). Fisher and McCarthy (2013) have provided a detailed list of key elements for informed consent that can guide development of concise summaries for prevention research involving genetic testing. Some of the key elements they identify include (1) how and for how long genetic material will be stored; (2) if when and how materials will be destroyed; (3) confidentiality protections including de-identification and risks of identity linkage; (4) the nature of personal genetic information that will or will not be disclosed to participants and the rationale for disclosure decisions; (5) opportunities for and limitations on the right to withdraw data once it has been collected, stored, or analyzed; and for pediatric research, (6) parental permission and child assent procedures, plans at the time child participants become legal adults; and (7) the possibility that data may contradict assumed attribution of paternity or other biological bases of family relationships.

## Transparency Requirements for Consent Forms Related to Clinical Trials

Maintaining high standards of transparency in representing themselves to stakeholders is a key recommendation from the SPR Ethics Task Force (Leadbeater et al. 2018). A significant change to the Common Rule that will have implications for prevention science is the new, broader definition of a “clinical trial” and transparency requirements for online posting of the consent forms for such trials online. The current proposed change in regulations defines a clinical trial as “a research study in which one or more human subjects are prospectively assigned to one or more interventions (which may include placebo or other control) to evaluate the effects of the interventions on biomedical or behavioral health related outcomes” §__.102(b) (OHRP 2017, p. 7260). Many social-behavioral scientists expressed concern that the absence of a clear definition of “intervention” inappropriately places non-intervention social and behavioral research involving manipulation of variables under the “clinical trial” umbrella and subject to these transparency requirements. In response to these concerns, Congress passed an omnibus bill (“Consolidated Appropriations Act, H.R. 1625,” 2018) delaying the implementation of the new clinical trials definition for projects that historically do not fall under the clinical trials definition until it can undergo more thorough review and consultation (Society for Research in Child Development (SRCD) 2018). However, the National Institute of Health interprets the new bill to refer only to prior approved studies and therefore is instructing primary investigators for all new submissions to submit their study as a clinical trial if it corresponds to the regulatory definition. As a result, investigators conducting prevention trials may be required to post one version of the informed consent form online at a specified federal website within 60 days from the close of participant enrollment. To date, no additional guidance was provided identifying the online options for investigators, although readers may refer to the Food and Drug Administration (FDA) ClinicalTrials.gov as a potential model for what may be required.

The advantages and disadvantages of applying the FDA model designed for pharmaceutical and medical research to prevention science will need to be determined as compliance with this regulation moves forward. Some authors have argued that increased transparency through an online posting of informed consent forms may facilitate the development of clear and comprehensive documents that reflect the spirit of the regulations’ focus on comprehension and decision-making (Bierer et al. 2017). However, this assumes that once such documents begin to be posted online, investigators will refer to these documents as a resource when developing their own consent process. More likely, the posting of consent forms will lead to greater transparency of the scope of intervention research, engendering expectations by the public and scientific community to examine study findings. This, in turn, may lead to pressure on prevention scientists to report null results, public pressure previously applied to pharmaceutical clinical trials (Fisher 2006a).

## Broad Consent

The rapid increase in biospecimens repositories and the ability to link this data with integrated administrative datasets is continuing to influence how potentially identifiable participant information and biospecimens are collected and shared across research, healthcare, educational, and criminal justice systems. At the potential cost of individual privacy, aggregating data requires identifying individuals across datasets and has several benefits. Often undetected by smaller, lower powered studies, large aggregated datasets provide a cost-effective method to identify complex associations between biology and environmental risk factors for poor outcomes. However, these integrated datasets require additional confidentiality protections both because researchers may need to utilize legitimate means of identifying and linking an individual’s information across data sets, and the increasing sophistication of individuals within and outside the research community to re-identify assumed protected de-identified information (Fisher 2017c; Homer et al. 2008; Lunshof et al. 2008). The introduction of broad consent in federal regulations arose in response to these concerns as well as recognition that additional participant protections were required for the long-term secondary use of potentially identifiable information by investigators who were not involved in the original collection of data and who might use the data for research purposes significantly different from the original study to which participants consented. Prevention scientists planning to store and maintain potentially identifiable information and biospecimens for future use by other researchers may substitute broad consent for the traditional consent procedures as long as the broad consent includes the components described in the next section and the reasonable person standard discussed above.

### Background and Rationale: Balancing Privacy Concerns with Advancing Technologies

How to protect the autonomy of participants without hindering an important avenue of research and discovery has been vigorously debated in recent years (Berkman et al. 2017; Dickert et al. 2017; Grady et al. 2015; Grady 2017). Under previous regulations, investigators wishing to use secondary data could re-consent participants from the original study or, more commonly, petition the IRB to waive consent for secondary use (OHRP 2017). The Final Rule recognizes that re-consent is potentially a costly, burdensome, and prohibitive process which may not only derail promising scientific advances but may introduce unnecessary risk of privacy violations during re-identification procedures. However, the new regulations also attempt to address instances in which waiver of consent for secondary analysis may lead to similar privacy risks and potential violations of participant autonomy. Broad consent is a regulatory attempt to achieve an appropriate balance between participant rights to determine the future use of their research data and the scientific benefits that may accrue when such use involves unspecified investigators and research aims.

#### Definition and Overview of Broad Consent Requirements

According to the new regulations, as part of the initial consent procedures, investigators may seek broad consent for the storage, maintenance, and secondary research use of identifiable private information or identifiable biospecimens collected for studies other than the initially proposed research or for non-research purposes (§__.116(d); (OHRP 2017, p. 7265). When compared to the practice of obtaining a waiver of re-consent for secondary analysis, broad consent increases transparency and provides greater opportunities for participants to decide if their identifiable private information or identifiable biospecimens may be used by future researchers for specified or unspecified research purposes (Menikoff et al. 2017). When broad consent is obtained, future research covered by the broad consent may be subject to limited IRB review designed only to determine if the proposed research is within the scope of the broad consent. IRB waiver of consent to use previously collected data for secondary use remains an option under the Final Rule. However, if broad consent has been offered but refused, an IRB cannot waive consent for secondary research use of that person’s identifiable private information or identifiable biospecimens.

The regulatory vision for broad consent for future research hinges on the extent to which participants are provided with details of the nature, storage, maintenance, and future uses of their identifiable data needed for the reasonable person to make an informed decision. To be sufficiently robust, the regulations mandate a series of information disclosures related to the type of data stored, time period for storage and use, types of future use, and with whom data may be shared. Mandates also include additional disclosures related to whole genome sequencings, clinically relevant data, and when data may be de-identified or glean commercial profit. A discussion of the required disclosures is provided below.

#### Definition and Description of Identifiable Information or Biospecimens

A significant change in the Final Rule is the inclusion of “identifiable information and biospecimens” under the definition “human subject” (§__.102 (e)1 (ii))(OHRP 2017). This expanded definition has been quite controversial since it means that identifiable information and biospecimens require the same protections as persons. Since the Final Rule requires investigators to describe the identifiable information and biospecimens that will be collected and stored for future use, it is noteworthy that the term identifiable is defined as “private information [or biospecimen] for which the identity of the subject is or may readily be ascertained by the investigator or associated with the information [or biospecimen]” (§__.102 (e) 5–7) (OHRP 2017, p. 7260). The inclusion of “may readily be ascertained” is in recognition of the evolving ability to re-identify previously de-identified genetic information. At present, what qualifies as an identifiable biospecimen is left to the discretion of the IRB, although the Final Rule includes a provision for convening a committee for periodic review of the definition. As a result, prevention scientists will need to keep abreast of continuously changing definitions of identifiable information and biospecimens for broad consent procedures. To date, the definition of socio-demographic identifiable information attached to biospecimens or as part of administrative systems linkages draws from the Health Insurance Portability and Accountability Act (HIPAA) and includes but is not limited to: name; place of birth and mailing address more specific than state residence; hospital admission or discharge date more specific than year; email, telephone, and fax; and Social Security or health plan number (U.S. Department of Health and Human Services 2012). The original participant ID number is also considered an identifier and will need to be re-coded if data is defined as de-identified.

#### Time Period for Storage and Use

A second required component is the time period of storage and use of the identifiable data, which can be limited or in perpetuity. Determining and communicating a time period for data use is particularly relevant when working with members of American Indian Alaskan Native (AIAN) nations. Some tribal communities, such as the Havasupai, prohibit body fragmentation and biospecimens living outside the body or after death (Bardill and Garrison 2015; Pearson et al. 2014; Sahota 2014). Broad consent for AIAN populations should, therefore, consult with AIAN leaders and carefully specify conditions for storage, destruction, and blessing before destruction of biospecimens (Arbour and Cook 2006). Extended time periods for storage also have implications for biospecimens collected on minors with guardian permission. Investigators must consider whether child participants when they become legal adults will be notified as to where their biological materials are stored and conditions in which they will or will not have a right to re-consent or withdraw permission for further use of their data (Fisher et al. 2013). When unlimited time periods for data storage and use conflict with cultural values or rights of minors, investigators may consider a model of “DNA on loan” where donations remain the property of the participant who gift their biospecimen to researchers who agree to act as faithful stewards for a specific period of time and for a specific research project (Arbour and Cook 2006).

#### Disclosure of Future Users

Broad consent must disclose who may have access to a participant’s stored identifiable data or clearly indicate that the identity of those with access to secondary use will remain unspecified. Most commonly, access will be limited to investigators affiliated with accredited universities, research, or medical institutions that have the institutional oversight and infrastructure to adequately protect data security and abide by restrictions on use outlined in the broad consent. When considering whether to conduct secondary research or to deposit data into biobanks or other data repositories, prevention scientists should investigate whether the repository conforms to applicable regulations and policies. The University of California (2012) has an informative guide to evaluating biorepositories that includes but is not limited to procedures for (1) identifying and ensuring that the aims of secondary usage requests are consistent with consent obtained from participants; (2) evaluating the qualifications of researchers and entities requesting access to data; (3) updating data security in response to emerging technologies; (4) including a submittal agreement from the original investigator attesting to the IRB approval and written informed consent of participants from whom data was collected; (5) providing a standard usage agreement that details conditions for receipt and future use of data or human specimens; and (6) continuing committee review and oversight.

#### Commercial Use of Data

The movement toward open science including the availability of data from prevention trials to other scientists and stakeholders (Caulfield et al. 2012; Leadbeater et al. 2018) and growing interest in genetic responsivity to psychopharmacological medications for behavioral and other mental health disorders is likely to be paralleled by increased interest in secondary use of biobank data by pharmaceutical companies and in funding prevention scientists to conduct such research. As recommended by the SPR Ethics Task Force (Leadbeater et al. 2018), investigators need to disclose financial and professional conflict of interests to all stakeholders, especially when presenting the scientific findings to stakeholders that affect program adoption, dissemination, and implementation strategies.

The new broad consent regulations require disclosure to participants if their biospecimens may be used for commercial profit and whether they will receive any portion of these profits. To be in compliance, at the outset of commercial sponsor funding relationships, investigators need to (1) reach an agreement on where data will be stored; (2) ensure data repositories meet current standards of informational security if deposited at a company facility; (3) have a clear understanding of which entity owns the data for future use; (4) whether such use will be for commercial profit; and (5) and whether participants will receive any portion of these profits.

#### Disclosure of Future Use

A fourth requirement of broad consent is a mandate to disclose how participants’ identifiable information may be used, whether whole genome sequencing may be conducted by other investigators, whether participants will be informed of future use, and if they are being asked to agree to future research that may not be aligned with the purpose of the original study. Although investigators are not required to return individual genomic results to participants, survey research indicates that genetic testing serves as a strong incentive for participation and individuals often prefer to be provided with incidental results that go beyond the original aims of the study (Kaufman et al. 2016). Ethical obligations for disclosure of clinically relevant genetic information uncovered during secondary analysis will increase as genetic research advances to adequately identify and successfully intervene to reduce vulnerability or enhance development for critical outcomes such as academic achievement, health, and well-being (Appelbaum et al. 2014; Fisher 2006b; Grandjean and Sorsa 1996; Jarvik et al. 2014; Ravitsky and Wilfond 2006). Broad consent must thus include a statement regarding whether participants will be informed of clinically relevant findings that may emerge.

### Participant Perspectives

Emerging empirical evidence on public willingness to provide broad consent or donate genetic information to biobanks for future research suggest that a majority of people are willing to consent for altruistic reasons if adequate privacy protections are in place, they are informed about who will use the data, and have a say in how their information will be used (Burstein et al. 2014; Hens et al. 2011; Kaufman et al. 2008; Kaufman et al. 2016; Lemke et al. 2010; McGuire et al. 2008; McGuire et al. 2011; Oliver et al. 2012). However, willingness to consent varies by the type of future research and the background of the prospective participant. According to one survey study, participants willing to donate biospecimens for future disease-related research were more hesitant on its use for socially sensitive topics such as such as abortion, genetic influences on violence, or vaccines related to biological weapons (De Vries et al. 2016). In a survey on adults’ opinions about a nationwide precision medicine initiative involving collection of genomic and environmental information, fewer respondents were willing to agree to the use of their data with researchers outside of the USA or pharmaceutical or drug companies than with American academic researchers or NIH researchers (Kaufman et al. 2016).

The perceived vulnerability of participants has also been shown to influence willingness to share of DNA samples for future research. In several studies, parents providing broad consent for their minor child expressed concern about unknown future risks and future decision-making for themselves and for their children, when their children become adults (Burstein et al. 2014; Hens et al. 2011; Kaufman et al. 2008). In one of the few studies examining youth attitudes, adolescents who were receiving outpatient oncology, cardiology, and orthopedics services were more willing to donate their specimens to biobanks than their parents or their healthy peers (Kong et al. 2016). In a large nationally sponsored epidemiological study on mental health and substance use, although donation rates were high overall, African American and individuals with less education and a history of drug abuse were less likely to consent to sharing their sample with other investigators (Storr et al. 2014). Attitudes toward collection, storage, and use of biospecimens may also differ by culture and social risk factors. For example, community members in Africa, Asia, and the America’s involvement in HIV prevention trials indicated that in some instances, sexual partners and spouses of participants wanted information on where biospecimens would be stored and to be included in decision-making (Mac Queen and Alleman 2008).

The public response to the expansion of genetic explanations for substance use, behavioral disorders, racial/ethnic differences in mental health, and other socially stigmatized behaviors has the potential to perpetuate health disparities by attributing vulnerabilities intrinsically tied to social and structural inequities to genetic characteristics (Fisher et al. 2013; Fisher and McCarthy 2013). The extent to which unspecified secondary use of biospecimens can pose a social risk to already vulnerable populations is difficult to anticipate or describe in broad consent procedures. Because participants may be unwilling to provide a blanket broad consent for future use of their specimens, prevention scientists may consider allowing participants to opt out of use of their data for specific future research purposes. This approach may, however, place unrealistic demands on researchers to identify all possible future uses and if not adequately specified place an unachievable burden on IRBs to interpret whether proposed secondary research aims meet the original broad consent specifications. One solution is to engage community advisory boards in creating explicit opt-out procedures tailored to the unique characteristics of participant populations to create a goodness-of-fit between broad consent procedures and participant values and concerns (Fisher 2015; Fisher and Ragsdale 2006).

## Conclusion

Researchers seeking broad consent are tasked with specifying the terms of the consent and engendering trust that the investigators, their institutions, and future researchers will be faithful stewards of participants’ identifiable information and biospecimens. Until the Final Rule is widely applied and tested, prevention scientists who utilize broad consent will face the challenging task of identifying potential future uses of data collected and determining the degree of specification necessary for the reasonable person to make an informed broad consent decision. Furthermore, investigators need to consider whether adopting an open-ended, broad consent for future use of identifiable data may discourage research participation or engender unreasonable expectations regarding future return of results. Investigators will also need to determine how to specify essential details without creating scripted templates for informed consent that do not adequately fit the participant’s health and genetic literacy. An additional responsibility falling on prevention scientists during this preliminary period is the need to determine whether their data repositories have the infrastructure to honor obligations made during broad consent. Infrastructure obligations include the ability to identify which participants in the original study refused broad consent to ensure their data is withheld from secondary research efforts, to enforce the limits promised on data access and the purpose to which the data will be used, and the destruction of biospecimens on the specified schedule promised during broad consent.

IRBs and their institutions face additional challenges. Policies will need to be developed that adequately determine if secondary researchers have the training and expertise to be a proper steward of participant protections for the use of identifiable information and biospecimens and can honor the obligations in the original broad consent. To support ongoing stewardship of identifiable genetic material, the SRCD Committee on the Common Rule recommended that institutions should develop procedures to ensure that researchers who have access to data in the future will be bound by the best practices in confidentiality protections at the time data was collected and new protections as they emerge (Fisher et al. 2013). As prevention scientists’ grapple with the current ambiguity and evolving interpretations of the Final Rule, an ethical awareness of the values and preferences with which participants approach the future use of their personal data will produce informed consent procedures that minimize informational risk, optimize participants’ informed choice, and promote prevention science.
